# Pharmacological chaperone reshapes the energy landscape for folding and aggregation of the prion protein

**DOI:** 10.1038/ncomms12058

**Published:** 2016-06-27

**Authors:** Amar Nath Gupta, Krishna Neupane, Negar Rezajooei, Leonardo M. Cortez, Valerie L. Sim, Michael T. Woodside

**Affiliations:** 1 Department of Physics, University of Alberta, Edmonton, Alberta, Canada T6G 2E1; 2 Division of Neurology, Department of Medicine, Centre for Prions and Protein Folding Diseases, and Neuroscience and Mental Health Institute, University of Alberta, Edmonton, Alberta, Canada T6G 2M8; 3 National Institute for Nanotechnology, National Research Council, Edmonton, Alberta, Canada T6G 2M9

## Abstract

The development of small-molecule pharmacological chaperones as therapeutics for protein misfolding diseases has proven challenging, partly because their mechanism of action remains unclear. Here we study Fe-TMPyP, a tetrapyrrole that binds to the prion protein PrP and inhibits misfolding, examining its effects on PrP folding at the single-molecule level with force spectroscopy. Single PrP molecules are unfolded with and without Fe-TMPyP present using optical tweezers. Ligand binding to the native structure increases the unfolding force significantly and alters the transition state for unfolding, making it more brittle and raising the barrier height. Fe-TMPyP also binds the unfolded state, delaying native refolding. Furthermore, Fe-TMPyP binding blocks the formation of a stable misfolded dimer by interfering with intermolecular interactions, acting in a similar manner to some molecular chaperones. The ligand thus promotes native folding by stabilizing the native state while also suppressing interactions driving aggregation.

## Abstract (web summary)

Small-molecule chaperones that can prevent protein misfolding have potential applications for treating diseases such as Alzheimer's and ALS. Here the authors use high-resolution force spectroscopy to gain insight into the mechanism of action of an iron-tetrapyrrole with anti-prion properties.

## Graphical abstract

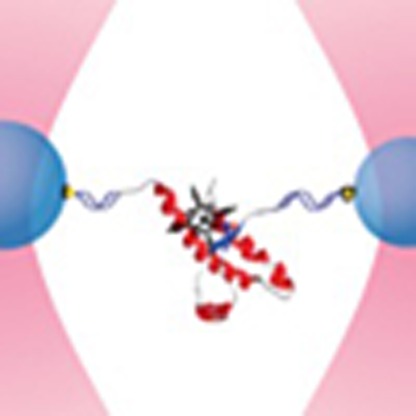

Misfolded proteins are an important feature of many neurodegenerative diseases, from Alzheimer's and Parkinson's to amyotrophic lateral sclerosis (ALS) and prionopathies, collecting in characteristic amyloid plaques^1^,^2^. Misfolding is normally held in check in the cell through the action of molecular chaperones, which help proteins find their native structure, preventing misfolding in the first place^3^, or the proteasome, which degrades incorrectly folded products^4^. However, this proteostatic machinery is likely overwhelmed in misfolded diseases^5^,^6^, allowing misfolded protein species—including the prefibrillar oligomers thought to be the most neurotoxic species^1^,^7^,^8^—to accumulate. Such a picture has motivated the development of small-molecule drugs that could act as pharmacological chaperones to promote native folding of disease-related proteins^9^,^10^. Such strategies have yielded a number of compounds with promising potential^11^,^12^, but it has proven challenging to improve their performance and develop effective therapeutics, in part because the mechanism of action of putative pharmacological chaperones is not known.

Single-molecule methods such as fluorescence and force spectroscopy provide a powerful new approach for addressing this question, because their ability to detect rare and transient states, identify and characterize different subpopulations in a heterogeneous ensemble, and follow conformational changes in a single molecule with high resolution^13^ is ideally suited to probing misfolding processes^14^,^15^. Single-molecule approaches have been deployed successfully to study protein misfolding and aggregation, for example identifying misfolded states, determining misfolding pathways, detecting transient oligomeric intermediates and exploring the interactions stabilizing amyloid fibrils^7^,^16^,^17^,^18^,^19^,^20^,^21^,^22^. They have also started to be applied to unravel the mechanisms of molecular chaperones^23^, showing for example that chaperones help correct folding of substrate proteins by unfolding misfolded molecules to give them a new chance to refold, altering the folding rates of domains, and blocking tertiary contacts in the transition state^23^,^24^,^25^,^26^,^27^. However, there has been little single-molecule work to date on pharmacological chaperones, aside from studies of their effects on amyloid stability^22^. Here we use single-molecule force spectroscopy (SMFS), wherein a single molecule is held under tension by an applied load and its extension is measured as its structure changes in response to the load^28^, to investigate the effect of a ligand with anti-prion activity on the folding of the prion protein PrP.

Misfolding of PrP causes prion diseases such as Creutzfeldt–Jakob disease, scrapie and bovine spongiform encephalopathy. The native, cellular form of PrP, rich in α-helices and denoted PrP^C^, is converted into a toxic, β-rich form, denoted PrP^Sc^, which has the ability to recruit further PrP^C^ molecules and thereby propagate the disease^29^,^30^. The structure of PrP^Sc^ remains controversial^31^,^32^,^33^, as does the molecular mechanism of the conversion of PrP^C^ (refs 2, 30). Despite these uncertainties about the central aspects of the molecular basis for prion diseases, however, several putative small-molecule chaperones with anti-prion activity have been discovered using cellular and/or animal models of disease^34^,^35^. Examples include sulphonated dyes such as congo red and its derivatives (for example, curcumin),^36^,^37^ certain polyanions^38^,^39^, 2-aminothiozoles^40^ and various heterocyclic compounds^41^,^42^,^43^,^44^,^45^. Notable examples of the latter include cyclic tetrapyrroles^11^,^46^ such as phthalocyanines and porphyrins, which have been found to inhibit PrP^Sc^ accumulation in cell culture^46^,^47^ and protein misfolding cyclic amplification assays^47^, as well as to increase the survival times in animal models^11^,^48^. However, the mechanism of anti-prion action has not yet been determined for any of these molecules.

Since deciphering how such ligands work could provide clues to the molecular mechanism for conversion of PrP^C^ into PrP^Sc^ and help design improved drugs, we investigated the effects of ligand binding on individual PrP molecules using SMFS. SMFS has previously been used to characterize the native folding pathway of PrP, measuring the folding energy landscape and hence properties of the transition state^49^, as well as to discover misfolding pathways available to PrP that might lead to aggregated structures^17^, reveal the sequence of steps leading to stable misfolded dimeric forms^21^, and probe the properties of the monomeric units comprising amyloid fibrils^50^. However, it has not yet been used to study the effects of an anti-prion ligand binding to PrP. Here we present the first such study, probing the effects of the anti-prion ligand iron(III) *meso-*tetra (*N*-methyl-4-pyridyl-porphine), hereafter denoted Fe-TMPyP, on the unfolding of PrP at the single-molecule level. Fe-TMPyP is known to bind to PrP with 1:1 stoichiometry and a dissociation constant of 11±1 μM, in a binding pocket located at one end of the molecule where it interacts with strand 1, helices 2 and 3, and the loop between strand 1 and helix 2 (Fig. 1a)^47^. We find that it acts in multiple ways, both stabilizing the native structure and inhibiting the formation of intermolecular interactions that stabilize misfolded aggregates.

## Results

### Force-extension curves of PrP

**Figure 1:**
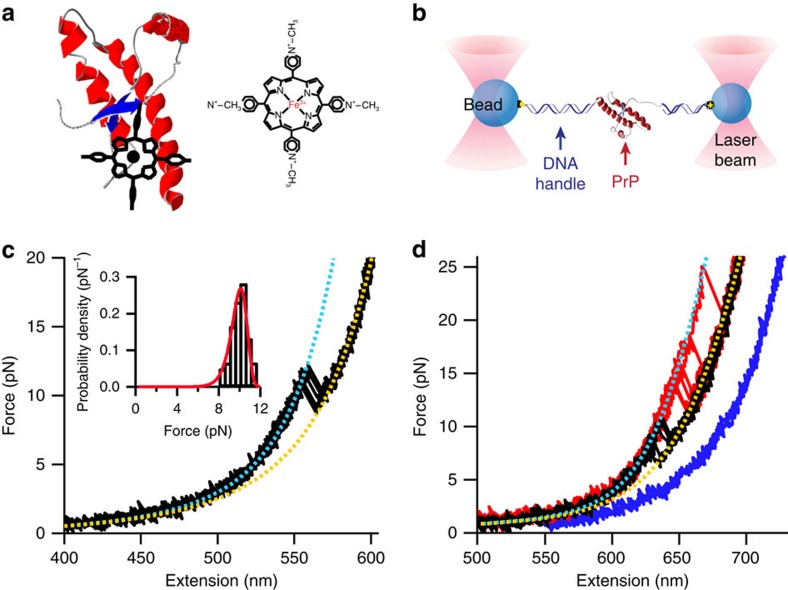
Force spectroscopy of PrP with Fe-TMPyP. (**a**) Structure of Fe-TMPyP (right). Fe-TMPyP binds PrP^C^ in a pocket as shown on left, interacting with the C terminus of helix 3, N terminus of helix 2, the helix 2-strand 2 loop and strand 1 (model based on ref. 47). (**b**) Schematic of force spectroscopy assay. PrP was attached covalently to DNA handles linked to polystyrene beads held by laser beams. (**c**) FECs in the absence of Fe-TMPyP (black), fit by WLC models for the folded (cyan) and unfolded (yellow) states, show a single unfolding event with a narrow unfolding force distribution (inset; *N*=200) peaked near 10 pN. Red line: fit to equation 3. (**d**) With 50 μM Fe-TMPyP, three types of FECs were observed, reflecting different states of the protein: natively folded but ligand-free (black), natively folded but ligand-bound (red) and ligand-bound but native structure disrupted (blue). Total number of FECs: 120. The unfolding force distribution for FECs with discrete transitions is much broader than without Fe-TMPyP.

Single molecules of hamster PrP were attached via kb-long DNA handles to polystyrene beads held in a dual-beam optical trap (Fig. 1b), as described previously^17^. In the absence of Fe-TMPyP, force-ramp measurements displayed a single type of behaviour: the force first rose non-linearly as the handles were stretched out, until there was a ‘rip' involving a sudden extension increase and concomitant force decrease (Fig. 1c), characteristic of cooperative unfolding of the protein. Notably, the unfolding occurred as a two-state transition, without any detectable intermediates^17^. We fitted the two branches of the force-extension curves (FECs) to the extensible worm-like chain (WLC) model for polymer elasticity (equation 1, Methods section) as described previously (Fig. 1c, cyan: folded, yellow: unfolded)^17^. The contour length change on unfolding, Δ*L*_c_, was 34.3±0.4 nm, matching the value expected from the NMR structure for hamster PrP^C^ as previously reported^17^. All unfolding events displayed similar Δ*L*_c_ values, and the distribution of unfolding forces, *p*(*F*_u_) (Fig. 1c inset), was that expected for a single unfolding barrier^51^, indicating that a single population of natively folded PrP was present.

When these measurements were repeated in the presence of 50 μM Fe-TMPyP, however, multiple populations with different behaviour were observed (Fig. 1d). Some unfolding curves (Fig. 1d, black) displayed similar average Δ*L*_c_ and *F*_u_ values to those seen without Fe-TMPyP: 〈Δ*L*_c_〉=34.8±0.5 nm and 〈*F*_u_〉=10.2±0.2 pN, once again using WLC fits to determine Δ*L*_c_ (Fig. 1d, dashed lines). These similarities indicate that this sub-population of molecules was natively folded, but there was no ligand bound and hence the unfolding behaviour was unchanged. Other curves (Fig. 1d, red) displayed the same Δ*L*_c_ as seen without Fe-TMPyP, 〈Δ*L*_c_〉=34.4±0.4 nm, but an average unfolding force that was significantly higher: 〈*F*_u_〉=15.6±0.5 pN. The similar Δ*L*_c_ indicates that the protein was still natively folded, but the increased *F*_u_ reveals that the structure was stabilised by the ligand. Finally, a third sub-population was observed: 25% of the curves did not contain any obvious unfolding rip (Fig. 1d, blue). In this case, the lack of a discrete unfolding event indicates that the ligand must have bound to the protein when it was unfolded at high force, in such a way as to prevent the formation of the native structure when the force was reduced to zero.

### Energy landscape for PrP unfolding

**Figure 2:**
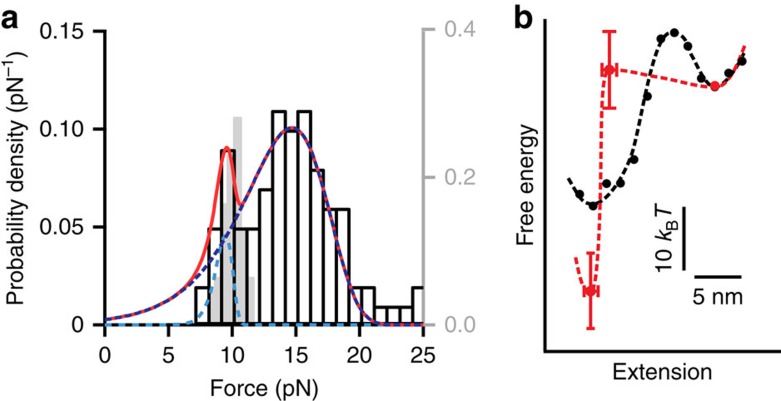
Effect of Fe-TMPyP binding on unfolding energy landscape. (**a**) The unfolding force distribution (black) for discrete transitions at 50 μM Fe-TMPyP had two peaks, near 9 and 15 pN (red: fit to equation 3). The low-force peak (cyan) matches the distribution for unfolding the native structure in the absence of ligand binding (grey; scale on right); the high-force peak (blue) corresponds to the unfolding of ligand-bound native structure. (**b**) Energy landscape for unfolding the native state without (black) and with (red) Fe-TMPyP bound.

To quantify the effects of ligand binding on the unfolding behaviour of the native structure, we analysed the distribution of unfolding forces for all FECs containing discrete rips. Unfolding force distributions for two-state transitions such as these have a well-defined, single-peaked shape, determined by the unfolding rate at zero force, *k*_0_, the height of the barrier for unfolding in the energy landscape, Δ*G*^‡^, and the distance to the barrier from the folded state, Δ*x*^‡^ (ref. 52). Whereas without Fe-TMPyP the force distribution (Fig. 1c, inset) displayed the single-peaked form expected from equation 3 (Methods section), with Fe-TMPyP present there were two peaks in *p*(*F*) (Fig. 2a), corresponding to two populations of natively folded PrP: ligand-free, with a peak near 10 pN (as in Fig. 1c, inset), and ligand-bound, with a peak near 15 pN. The distribution for ligand-free PrP^C^ has been studied in detail previously^49^; it is relatively narrow, indicating a ‘compliant' structure with an extended transition state that is quite sensitive to the application of force^52^. The distribution for ligand-bound PrP^C^ is clearly much wider, indicating that the structure has become more ‘brittle', with a more compact transition state that is less sensitive to force. Fitting the complete unfolding force distribution to two independent two-state transitions (Fig. 2a, red), one for each population (cyan: ligand-free, blue: ligand-bound), we found that indeed Δ*x*^‡^ decreased considerably on ligand binding, from 9±1 nm to 1.3±0.3 nm. In contrast, Δ*G*^‡^ measured from the native state increased to 36±7 *k*_B_*T* with the ligand bound, compared with 26±2 *k*_B_*T* when ligand-free.

The ligand-bound state represented 85±5% of the FECs with PrP natively folded. Assuming binding in equilibrium, this implies a dissociation constant *K*_d_=9±3 μM, comparable to the value 11±1 μM measured by isothermal titration calorimetry under comparable conditions^47^. Estimating the stabilizing effect of ligand binding from *K*_d_, we found ΔΔ*G*=−11.6±0.5 *k*_B_*T*. A complementary analysis using the Jarzysnki equality^53^ (equation 4) to estimate Δ*G* from the distribution of work done during unfolding yielded a similar result, ΔΔ*G*=−12±5 *k*_B_*T*. Combining the free energy change from ligand binding with the changes to the barrier properties, we reconstructed the effect of ligand binding on the energy landscape profile for native unfolding, illustrated in Fig. 2b (black: ligand-free; red: ligand-bound).

### Fe-TMPyP can hinder native folding

**Figure 3:**
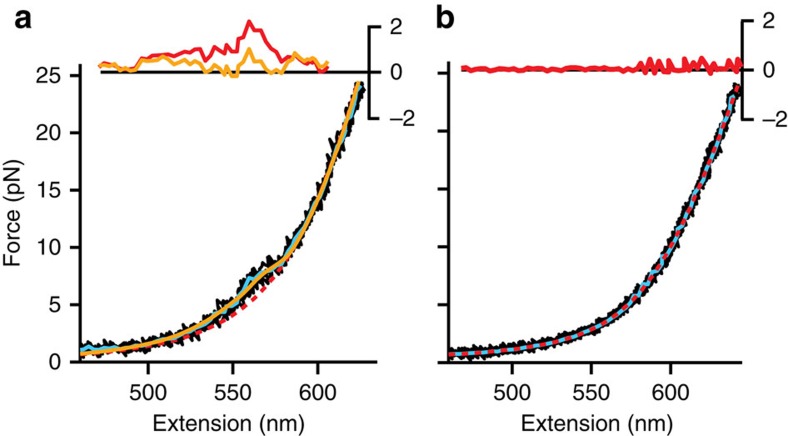
FECs without discrete transitions. (**a**) From ∼4–7 pN, these FECs are not well fit by the WLC model (red), leaving a systematic residual (inset). The average of these FECs (cyan) is well fit by a model (equation 2) with two misfolded states fluctuating in equilibrium (yellow). (**b**) FECs of the construct containing DNA handles only (black). Average of FECs (cyan) is fit very well by the expected simple WLC model, as evidenced by the lack of residual (inset), showing no shoulder feature.

Turning to the FECs not showing discrete unfolding transitions (Fig. 3a, black), if the protein is truly unfolded in these curves, they should fit well to the WLC model for a non-interacting polymer. Careful examination of these FECs, however, shows that they deviate from a pure WLC at low force (Fig. 3a, red dashed line): averaging the FECs to reduce noise (Fig. 3a, cyan), we find that there is a shoulder-like feature in the force range 4–7 pN, giving rise to a residual from fitting to the unfolded-state WLC (Fig. 3a, inset). Such features were previously observed for rapid but not very stable structural fluctuations in the intrinsically disordered protein α-synuclein^54^. In contrast, measurements of the DNA handles only show no such feature (Fig. 3b). The FECs without discrete unfolding events were compared with a model that added the force-dependent average extension of the fluctuating structures to the extension of the handles and unfolded protein (equation 2). Intriguingly, good agreement (Fig. 3a, yellow) was obtained by assuming that the rapid fluctuations involved the two rapidly forming misfolded states (labelled M1 and M2) observed previously in single PrP molecules^17^. These misfolded states are normally very short-lived (<1 ms) because they are much less stable than the native structure. Presumably they persist longer here because Fe-TMPyP binding to the unfolded chain suppresses native folding, at least temporarily.

These results show that the Fe-TMPyP binding affects the folding of a single PrP molecule on several levels. In addition to stabilizing the native structure, it also alters the nature of the transition state for unfolding PrP^C^, both raising the height of the barrier that must overcome to escape the native state and making the transition state more brittle. Such a picture matches what one would expect from previous work modelling the binding of Fe-TMPyP to PrP^C^ (ref. 47): the ligand interacts with both strand 1 near the N terminus of the structured domain in PrP^C^ and with helix 3 near the C terminus (Fig. 1a), hence it should act as a clamp making the transition state more native-like and thus reducing Δ*x*^‡^.

Whereas all of these effects suggest that Fe-TMPyP acts by stabilizing the natively folded state through multiple means (thermodynamically, kinetically and mechanically), the binding of Fe-TMPyP to the unfolded state complicates this picture. Although such binding has not previously been reported, it can be understood in the context of closely related porphyrins such as phthalocyanines, which are known to bind weakly to unstructured proteins such as α-synuclein^55^,^56^, including in particular the unstructured domain of full-length PrP (ref. 57), likely through interactions with aromatic residues^56^. The fact that binding of Fe-TMPyP to the unfolded state delays the formation of the native state would naively seem to contradict the anti-prion action of the ligand. However, binding to unfolded PrP might still play an important role in the context of aggregation, by preferentially blocking the formation of more stable misfolded oligomeric states, as proposed for α-synuclein^56^. Such an effect could be especially important for PrP, since PrP^Sc^ is an oligomeric form of the protein^30^.

### Effects on PrP dimers

**Figure 4:**
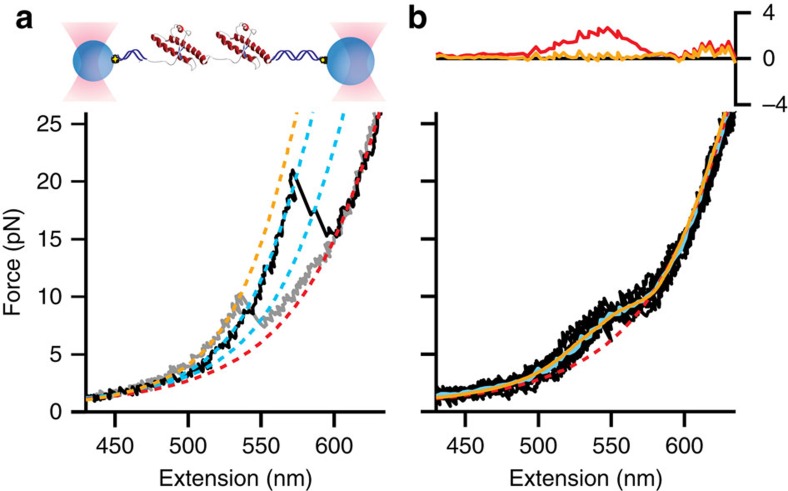
FECs of PrP dimer. (**a**) Inset: schematic of tandem dimer. FECs without Fe-TMPyP (grey) reveal a total Δ*L*_c_ more than twice the value for isolated monomers, indicating that the dimer forms a stable, non-native state. With 50 μM Fe-TMPyP (black), some FECs unfolded in two steps, each having the same Δ*L*_c_ as for unfolding PrP^C^, indicating that both domains were natively folded. Dotted lines: WLC fits (yellow, misfolded dimer; red, unfolded; cyan, natively folded domains). Total number of FECs: 164. (**b**) Most FECs with 50 μM Fe-TMPyP (black) showed no discrete transitions. The average (cyan) deviated markedly from a simple WLC model (red), but was well fit by the same model as for Fig. 3 (yellow). Inset: fit residuals.

To test this notion, we measured the effect of Fe-TMPyP on PrP dimers constructed by linking two PrP(90–231) domains in tandem (Fig. 4a, inset)^14^,^21^. Without TMPyP, the dimer invariably unfolded with Δ*L*_c_ values that did not match the native structure (Fig. 4a, grey), indicating that it was always misfolded, as reported previously^21^. This misfolded dimeric structure, labelled M_D_, was more stable thermodynamically than PrP^C^, with native structure never being observed in either domain of the dimer^21^. In the presence of Fe-TMPyP, however, the M_D_ state was observed only rarely. Instead, most FECs showed a shoulder feature without any sign of discrete transitions (Fig. 4b, black). Using equation 2, we found that a model based on the same parameters as in Fig. 3 was successful in replicating the full shape of the dimer FECs (Fig. 4b, yellow), but now with each monomeric misfolded state M1 and M2 occurring twice (once per monomer). Crucially, however, some FECs showed two discrete unfolding transitions, each with Δ*L*_c_ similar to that for unfolding native PrP^C^ (Fig. 4a, black). Fe-TMPyP was thus effective at preventing the formation of the stable misfolded dimer, thereby giving the less-stable native structure a chance to form, presumably assisted by the ability to bind to unfolded domains and in this way preferentially select against the non-native interactions driving the stable misfolding.

### *In vitro* aggregation

**Figure 5:**
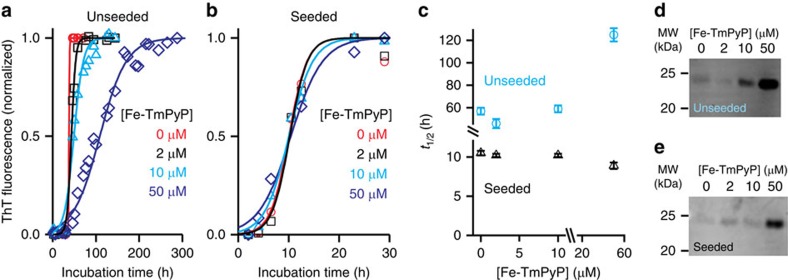
Effect of Fe-TMPyP on ensemble aggregation kinetics. (**a**) Time course of aggregation monitored by ThT fluorescence measured at different concentrations of Fe-TMPyP, without seeds present. Solid lines: fits to equation 5. (**b**) Same in the presence of seeds. (**c**) The time to reach half-maximal fluorescence was unaffected by Fe-TMPyP dose with seeds present, but was increased by a high dose in unseeded reactions. (**d**,**e**) The amount of soluble PrP not sequestered in fibrils increased significantly at high Fe-TMPyP doses for both (**d**) unseeded and (**e**) seeded reactions.

To confirm that Fe-TMPyP was able to interfere with the aggregation of our PrP constructs, we complemented the pulling studies with *in vitro* aggregation assays of PrP in the presence or absence of Fe-TMPyP (Fig. 5a,b), using thioflavin T (ThT) fluorescence to monitor the formation of ThT-positive aggregates under partial denaturing conditions^58^. Previous work used protein misfolded cyclic amplification to show that Fe-TMPyP can reduce prion amplification by half at a dose of 11 μM (ref. 47), but this study only quantified the final amounts of aggregated prion, with no assessment of the kinetics of aggregation. In unseeded reactions, the time to reach half-maximal ThT values, *t*_½_, was little changed by adding in low doses (2 and 10 μM) of Fe-TMPyP, but more than doubled with 50 μM (Fig. 5c, blue). This change in *t*_½_ was not accompanied by any significant change in lag phase duration, indicating that the primary effect of Fe-TMPyP was on the rate of fibril growth rather than seed formation. The total amount of insoluble aggregate was also reduced at 50 μM Fe-TMPyP, as measured from absolute fluorescence values and western blots of the supernatant (Fig. 5d), supporting the observation that 50 μM of Fe-TMPyP was able to reduce PrP aggregation. Repeating the aggregation experiments under seeding conditions by incubating monomers with pre-formed fibrils, the lag phase was greatly reduced compared with the unseeded reactions as expected (<6.5 versus >40 h), but no significant effect on *t*_½_ was detected (Fig. 5c, black). The amount of insoluble PrP aggregate was again reduced at 50 μM Fe-TMPyP (Fig. 5e).

## Discussion

**Figure 6:**
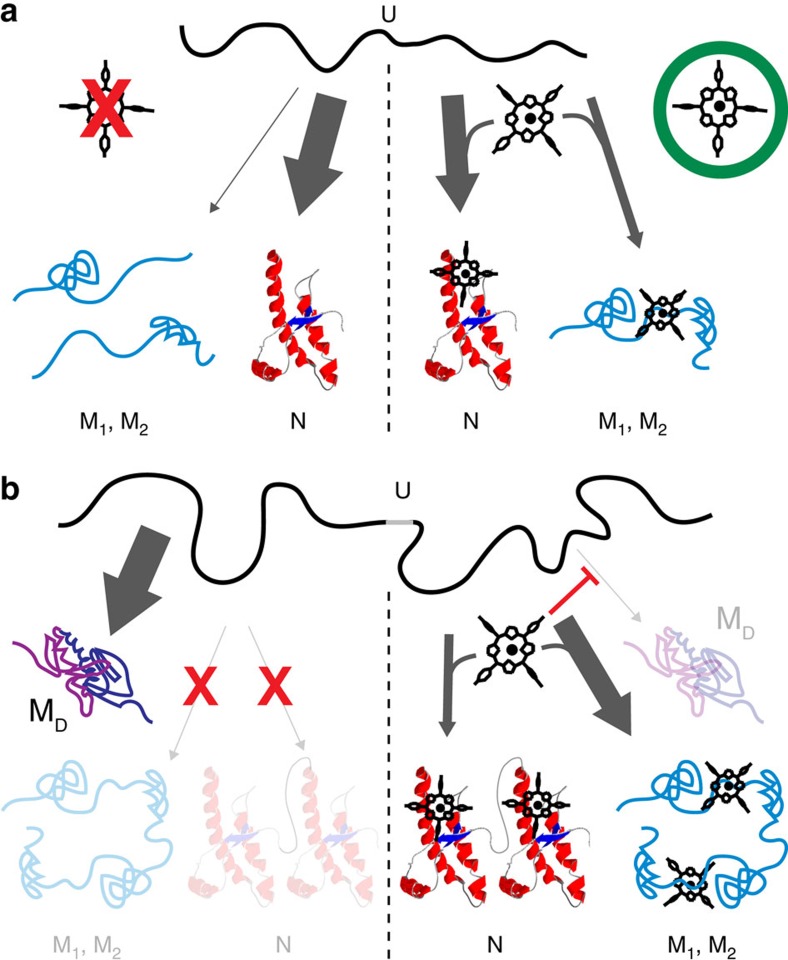
Cartoon of Fe-TMPyP effects on PrP folding. (**a**) Without Fe-TMPyP (left), PrP monomers fold natively; they can form misfolded states (for example, M1 and M2), but only transiently as they are unstable. With ligand (right), most of the time natively folded PrP is observed, but ligand binding to the unfolded protein can also allow misfolded states (M1 and M2) to form. (**b**) For dimers, without ligand (left) native folding is never observed, nor M1 and M2; instead, a stable misfolded dimeric structure forms. With ligand present (right), this stable misfolded structure is inhibited, and the structures observed in monomeric PrP are recovered (native fold or M1 and M2).

These results show that Fe-TMPyP binding does much more than simply stabilize the native state thermodynamically (an effect that does not truly represent a chaperone-like action): it also stabilises the native structure mechanically (by making it more rigid) and kinetically (by keeping the barrier energy roughly the same, so that the barrier for unfolding is increased because of the native state stabilization). The most interesting effects, however, relate to the ability of Fe-TMPyP to bind to PrP when unfolded, a property that was previously unsuspected. Such unfolded-state binding affects the folding of isolated PrP molecules, permitting the unstable misfolded states M1 and M2 to persist longer than they otherwise would. If these misfolded states M1 and M2 that are kinetically stabilised by the ligand binding were important on-pathway intermediates in the aggregation process, for example as nucleation points for forming seeds, then one might expect the unfolded-state binding of Fe-TMPyP to increase aggregation via reduced lag time. If, on the other hand, M1 and M2 are off-pathway, then the binding is likely to have only a minor effect: once the ligand unbinds, the misfolded states will rapidly refold into the native structure (as seen previously in the absence of ligand^49^). The measurements with dimeric PrP, which showed M1 and M2 but almost no M_D_ (in contrast to the case without ligand), suggest that the former are in fact off-pathway, and do not lead to more stable misfolded states. Furthermore, the fact that M_D_ is rarely seen with Fe-TMPyP present indicates that Fe-TMPyP binding must occur at a location that prevents the forming of M_D_. Given previous work identifying an intermediate (I_D_3) formed by the inter-domain region encompassing the C terminus of one monomer domain and the N terminus of the other as the first step on the pathway to forming M_D_ (ref. 21), and given that when Fe-TMPyP is present the signature of I_D_3 folding is seen only when M_D_ actually forms, Fe-TMPyP likely binds to the same inter-domain region involved in I_D_3 formation. By inhibiting M_D_ in this way, Fe-TMPyP binding creates an opportunity for native structure to form in the dimer (Fig. 6).

The picture of the effects of Fe-TMPyP at the single-molecule level, that blocking stable intermolecular contacts plays an important role, is consistent with the results from the ensemble aggregation assays. The assumption was previously made that if the compound could bind PrP^C^ and reduce prion formation, it was likely through an effect on the PrP^C^ monomer itself^47^. However, our results show that only at higher doses (50 μM) does Fe-TMPyP significantly alter the kinetics of PrP aggregation, via the growth rate rather than the lag phase. This means that Fe-TMPyP does not interfere with seed formation, but rather with the ability for seeds to promote further aggregation. In unseeded reactions, presumably the ability to bind seeds as they formed would significantly slow aggregation because the number of seeds forming would be small compared with the amount of Fe-TMPyP available. In contrast, the lack of effect on aggregation when the reaction was flooded with seeds suggests that the strength of interaction between Fe-TMPyP and seeds is relatively low and can be overwhelmed by excess seeds. Even in the seeded reactions, however, the effects of Fe-TMPyP can still be detected in the increased levels of residual non-amyloid PrP in the supernatant, analogous to the reduction of prion amplification by protein misfolded cyclic amplification.

It is interesting to compare these results for a pharmacological chaperone to the action of cellular chaperones. SMFS studies of the effects of trigger factor on the folding of maltose-binding protein (MBP) found that it stabilized partially folded intermediates rather than the native structure^24^, in contrast to the effect of Fe-TMPyP in binding and stabilizing PrP in its native structure. On the other hand, trigger factor also reduced interactions between domains in a tandem-repeat oligomer of MBP, thereby reducing the formation of stable misfolded states, very similar to the effect of Fe-TMPyP on PrP dimers. A second chaperone, secB, had a similar effect on tandem MBP oligomers, preventing stable aggregation, but secB bound primarily to the unfolded or molten-globule states, suppressing native folding as well^59^. A similar mechanism of action—preventing non-native inter-domain interactions—was also suggested by SMFS studies of the multi-domain protein luciferase^25^, as well as by fluorescence studies of huntingtin showing that the chaperone prefoldin suppresses the formation of toxic oligomers^60^.

Fe-TMPyP thus shares some of the characteristics of these cellular chaperones, particularly the suppression of inter-domain interactions leading to stable misfolded aggregates, but differs in its strong influence on the native state. In the context of prion disease, both of these effects might play a role in the anti-prion action of Fe-TMPyP, since the stabilization of the native state would tend to reduce the rate of conversion and the suppression of non-native inter-domain contacts would tend to reduce the growth rate of oligomers. However, the aggregation kinetics suggest that the latter may play the more important role, highlighting the similarities with cellular chaperones. Extending measurements like these to other anti-prion ligands and pharmacological inhibitors of misfolding in other proteins should allow commonalities in the molecular mechanisms of pharmacological chaperones to be identified, revealing new ways to develop improved drug candidates.

## Methods

### Sample preparation

Syrian hamster prion protein (SHaPrP) containing residues 90–231 (which form the protease-resistant fragment in PrP^Sc^) was engineered by adding Cys residues at each terminus and cloned into the pET-15b plasmid with a N-terminal histidine tag as described previously^17^. It was expressed in *E. coli* BL21(DE3) cells, and purified by FPLC (GE Healthcare) using a Ni-NTA column. The purity and identity of the protein were verified by SDS–polyacrylamide gel electrophoresis and western blotting (Anti-prion(109–112) clone 3F4, Millipore), and native folding was confirmed by circular dichroism spectroscopy. DNA handles were attached to the refolded protein after dialysis into 50 mM sodium phosphate buffer, pH 7.0, as described previously^17^,^61^. Briefly, the protein was reduced with *tris*(2-carboxyethyl)phosphine (TCEP) in a 100:1 molar ratio for 30 min, excess TCEP was removed using desalting spin columns, and the protein was activated with 2,2′-dithiodipyridine (DTDP). Retention of the native fold after activation was confirmed by circular dichroism spectroscopy. The activated protein was then reacted with sulfhydryl-labelled DNA handles prepared by PCR: one (798 bp) labelled by biotin, the other (1,261 bp) labelled with digoxigenin. We verified that the internal cysteines in PrP were non-reactive and that the handle functionalization was thus specific to the terminal cysteines by subjecting wild-type SHaPrP (which contains only the internal cysteines) to the same DTDP activation process as for the Cys-terminated PrP: mass spectrometry showed that wild-type SHaPrP did indeed not react with DTDP, in contrast to the Cys-terminated PrP.

SHaPrP dimers were made by cross-linking the sulfhydryl groups of the terminal Cys residues in monomers, as described previously^21^. Briefly, incomplete activation of the terminal Cys residues by DTDP produced PrP molecules with one Cys not activated by DTDP. The unactivated Cys on these molecules then reacted on oxidation with DTDP on a second PrP molecule to generate a dimer, to which DNA handles were attached in the same way as for monomers.

All PrP-DNA constructs were incubated at ∼100 pM with 250 pM polystyrene beads (600-nm diameter labelled with avidin, 800-nm diameter labelled with anti-digoxigenin) for ∼1 h at room temperature to form dumbbells^62^. Dumbbells were then diluted to ∼500 fM in the measurement buffer (50 mM MOPS, pH 7.0, 200 mM KCl, with an oxygen scavenging system consisting of 8 mU μl^−1^ glucose oxidase, 20 mU μl^−1^ catalase, and 0.01% w/v D-glucose) and inserted into a 5–10 μl sample cell cleaned by plasma etching, before being placed in the optical trap. Fe_TMPyP was added to protein during this last dilution stage right before measurement, to a final concentration of 50 μM. Because dumbbells were prepared at zero force under native conditions, the protein remained natively folded during the assembly process.

### FEC measurement and analysis

Each dumbbell was calibrated for position detection before measurement by measuring the detector voltages while raster scanning the beads through known positions^63^. FECs were measured by first holding the molecule at near-zero force for 5 s, to allow ligand binding, and then ramping the force up to ∼30 pN by moving the traps apart at a constant speed of ∼125 nm s^−1^. The force was then relaxed to ∼0 pN and the cycle repeated; each molecule was measured ∼20–30 times. Data were sampled at 20 kHz and filtered online at the Nyquist frequency. Note that large but uncalibrated forces that likely unfolded the protein were applied before FECs measurements could be made, hence there was no detectable difference between the properties of the first FEC and subsequent measurements of the same molecule.

FECs displaying discrete unfolding transitions were fit to an extensible WLC model relating the applied force, *F*, and molecular extension, *x*:


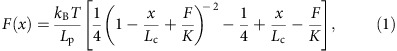


where *L*_p_ is the persistence length of the polymer, *L*_c_ is its contour length and *K* is the enthalpic elasticity. Two WLCs in series were used for the fitting, one to describe the DNA handles, the other to describe the protein^17^. The WLC parameters for the DNA handles, found from fitting the folded state of the FECs were *L*_p_∼40 nm, *L*_c_∼700 nm and *K*∼1,200 pN. The parameters for the protein, used to fit the unfolded state, were *L*_p_=0.65 nm, *L*_c_=0.36 nm per amino acid and *K*=2,000 pN (ref. 17).

FECs displaying shoulder features rather than discrete rips were compared with a model describing the extension change owing to the existence of rapid, quasi-equilibrium fluctuations that could not be directly resolved:^54^


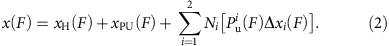


Here, *x*_H_(*F*) is the extension of the handles (obtained by inverting equation 1), *x*_PU_(*F*) is the extension of the unstructured portion of the protein, Δ*x*_*i*_(*F*) is the extension of a given structural fluctuation at a given force *F* and *P*_u_(*F*) is the probability of unfolding that structure at that force^64^. The index *i* represents different possible structures; the unfolding of each structure is characterized through the contour length change, Δ*L*_c_, and the force at which the structure is 50% likely to be unfolded, *F*_½_. Here, the structures were taken to be the same misfolded states of PrP that were observed in previous work, labelled M1 and M2 (ref. 17). The parameters for the structures were initially fixed to the values found previously^17^, so that there were no free parameters for fitting. On allowing the parameters to vary, there was no significant improvement in the fitting as revealed by three different tests: a sum-of-squares lack-of-fit test, the reduced *χ*^2^ and the Wald–Wolfowitz runs test^54^. *N*_*i*_ was fixed to 1 for monomer (one instance of each structure) and 2 for dimer data.

### Energy landscape analysis

Unfolding force distributions for PrP in the absence of Fe-TMPyP were fit to the theory of Dudko *et al*.^51^


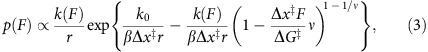


where 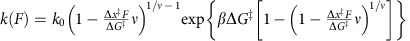,

*k*_0_ is the unfolding rate at zero force, Δ*x*^‡^ is the distance from the folded state to the barrier, Δ*G*^‡^ is the barrier height, *β*=1/*k*_B_*T* is the inverse thermal energy and *ν*=2/3 (representing a linear-cubic potential profile). Distributions of the unfolding forces in the presence of Fe-TMPyP for FECs containing discrete unfolding transitions were fit by a sum of two distributions represented by equation 3, one for the unbound state and one for the ligand-bound state. The parameters for the unbound state were fixed at the values found for the distribution in the absence of the ligand.

The free energy of binding to the native state was estimated from the distribution of work done during the FECs using the Jarzynski equality:^53^


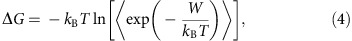


where *W* is the non-equilibrium work done to unfold the molecule. The Jarzynski estimate was corrected for the bias in the estimator^65^.

### Effect of instrumental artefacts on analysis

Recent work has begun to quantify the extent to which attaching molecules to force probes via compliant linkers as in SMFS can generate artefacts in the rates observed experimentally, slowing them down compared with the ‘intrinsic' rates that would be expected for isolated molecules^66^,^67^,^68^. We estimated the effect of these artefacts using the theory of Cossio *et al.*,^67^which requires knowledge of the fluctuations and autocorrelation decay time of the extension in the folded or unfolded state (available from extension trajectories at a given force^49^), the apparent Kramers rate determined from the measured kinetics and the observed potential of mean force, and the barrier height and curvature in the intrinsic molecular energy landscape (available from landscape reconstructions^49^). Using equations 7 and 13 from ref. 67, we found that the unfolding rate observed in the FEC measurements is estimated to be ∼30% lower than the ‘intrinsic' rate when unfolding ligand-bound PrP^C^, and ∼85% lower when unfolding ligand-free PrP^C^ (where the difference arises from the difference in Δ*x*^‡^).

The practical effect of these artefacts on our analysis is to increase the observed unfolding force above what would otherwise be expected, owing to the slower unfolding rate. We estimated the size of this force increase using the Bell–Evans–Zhurkov approximation, *k*(*F*)∝exp(*F*Δ*x*^‡^/*k*_B_*T*), for the force-dependence of the unfolding rate. The artefactually low unfolding rates would be expected to increase the force above the value expected from the intrinsic rate by ∼0.2 pN for ligand-free PrP^C^ and ∼0.3 pN for ligand-bound PrP^C^. Hence the rate artefacts cause the unfolding force to be overestimated by the same small amount in each case, ∼2%, and do not materially affect the analysis.

### Aggregation assays

200 μl of PrP at 0.5 mg ml^−1^ in 50 mM sodium phosphate buffer pH 7.0 and 2 M GdnHCl was placed in clear-bottomed wells of a 96-well plate (Costar 3610) covered with thermal adhesive sealing film (08-408-240; Fisherbrand). Samples were incubated at 37 °C with continuous shaking at 500 r.p.m. in the presence of 10 μM ThT. Fluorescence measurements were taken at 445/482 nm excitation/emission and 475 nm cutoff on a fluorescence plate reader (Molecular Devices, M5 Spectramax). At least three replicates were measured for each condition. For seeding experiments, the reaction was carried out in the presence of 0.00025% (w/v) pre-formed recombinant PrP fibrils. Experiments were run for 358 h for unseeded reactions and 47 h for seeded reactions.

The time course of the ThT fluorescence, *Y*(*t*), was normalized by setting the final ThT values to 1 and then fitted to


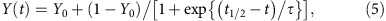


where *Y*_0_ is the initial fluorescence reading, *t*_1/2_ is the time at which the ThT fluorescence is half-maximal, and *τ* is the time required for the fluorescence to reach the final steady state^69^. We note that Fe-TMPyP partially quenched ThT fluorescence. To quantify the quenching, we added 2, 10 or 50 μM Fe-TMPyP to the products of unseeded control aggregation reactions and compared fluorescence readings before and after. The fluorescence values dropped upon Fe-TMPyP addition, respectively, to 99.1, 44.8 and 20.0% of the value for the control reactions. As an additional test of the amount of aggregation that occurred, we also quantified the amount of PrP that was incorporated into insoluble fibrils in the reactions, by centrifuging the end products after the aggregation assays were completed to remove insoluble fibrils and then immunoblotting the supernatants for residual PrP.

### Data availability

The data that support the findings of this study are available from the corresponding author on request.

## Additional information

**How to cite this article:** Gupta, A. N. *et al.* Pharmacological chaperone reshapes the energy landscape for folding and aggregation of the prion protein. *Nat. Commun.* 7:12058 doi: 10.1038/ncomms12058 (2016).
